# Effect of Lance Structure on Behavior of Coherent Jet in EAF Steelmaking Process

**DOI:** 10.3390/ma13051043

**Published:** 2020-02-26

**Authors:** Fuhai Liu, Rong Zhu, Guangsheng Wei, Shiliang Fan

**Affiliations:** 1 National Center for Materials Service Safety, University of Science and Technology Beijing, Beijing 100083, China; 2 Key Laboratory of Fluid Interaction with Material, Ministry of Education, University of Science and Technology Beijing, Beijing 100083, China; zhurong1206@126.com; 3 Xining Special Steel Co., Ltd, Qinghai, Xining 810005, China; fanman2006@126.com

**Keywords:** coherent lance, electric arc furnace, numerical simulation, combustion experiment

## Abstract

During the electric arc furnace steelmaking process, the coherent jet technology was widely used to protect the kinetic energy of the supersonic oxygen jet and achieve better mixing effects. Comparing with the conventional oxygen lance, the coherent lance could increase the surface area of impaction cavity, resulting in a better stirring effect and higher reaction rate. However, there was limited research about the effect of restriction structure for the coherent lance tip on the flow field characteristic of the main oxygen jet. In this research, three kinds of restriction structures have been investigated by numerical simulation and combustion experiment at room and high ambient temperature conditions. Then an optimum restriction structure would be tested in a 75 t electrical arc furnace steelmaking process to verify its metallurgical property.

## 1. Introduction

The oxygen lance plays an important role in the electric arc furnace (EAF) and basic oxygen furnace (BOF) steelmaking processes, which delivers the oxygen into the molten bath under supersonic condition [1,2]. Before the supersonic jet reaches the molten bath, its velocity keeps reducing, because of an entrainment phenomenon between the jet and the surrounding ambient gas, which lowers its impaction ability and oxidation rate [3,4]. To suppress this phenomenon, a shrouding gas flow has been adopted to reduce the environmental resistance for the supersonic oxygen jet. The shrouding gas flow, including gas/liquid fuel and oxygen, forms a combustion flame within the high-temperature environment of the furnace, and then a low-density zone is generated at the center of the flame. As a result, the potential velocity core for the supersonic oxygen jet will be prolonged, and this method to maintain the velocity of the supersonic oxygen jet is defined as the coherent jet technology, and the oxygen lance using the coherent jet technology has been defined as the coherent oxygen lance [5,6,7]. Nowadays, the coherent jet technology is widely used in the EAFs to control the oxygen supply process, and its metallurgical and operational benefits have been well reported [8,9].

As coherent lance is one of main apparatuses used in the EAF steelmaking process, there has been a lot of research regarding the effect of the shrouding nozzle arrangement, the process control, and the initial working parameters on the behavior of the coherent jet. During the research process, the application of coherent lances is hard to analyze by water experiment or multi-phase flow simulation, because these methods do not easily reproduce the combustion process. Therefore, the numerical simulations, combustion experiments, and industrial applications are always used to analyze the flow field characteristic of the coherent jet [10,11]. There are two main methods which are currently adopted to improve the stirring ability of coherent jets. The first one involves augmenting the total energy by adding more methane or by increasing the initial temperature of the input gas flow. The second one is optimizing the arrangement for shrouding nozzles, or selecting an appropriate characteristic curve for the Laval nozzle to suppress the generation of the shock waves for the main oxygen [12,13].

Liu et al. [14] have designed a shrouding nozzle using a supersonic structure for the coherent lances by means of simulation and experimental research, and the result showed the supersonic structure used in this method improved the potential core length. Odenthal et al. [15] has examined the development of coherent jet technology for EAF processes, and has analyzed the advantages of having an SIS (Siemag Injection System) coherent lance in terms of reducing the natural gas consumption and increasing supersonic oxygen jet impaction ability. Klioutchnikov et al. [16] had analyzed the flow field of coherent jet in hot-temperature environment, and achieved a formula to describe the length of velocity potential core for the coherent jet when the Mach number for both the main and the shrouding jet was same. Hu et al. [17] has proposed a mixed injection method for the shrouding jet to reduce its methane consumption, and has specified an appropriate mixing rate for N_2_ and CH_4_ in the control process. However, few studies have examined how to design an effective structure to channel the main and shrouding jet for increasing the impaction ability of the coherent jet, after they passed through the nozzles.

In this research, we first determine the positioning and flow rate for both the main and shrouding jets. Then, we proposed three kinds of the restriction structures at the coherent lance tip, and compare their performance to a conventional lance (supersonic oxygen lance) by a series of numerical simulations and combustion experiments. The axial velocity and static temperature of the main oxygen jet at the centerline are measured in combustion experiment, to verify the accuracy of the simulation data. Based on the results, a proper restriction structure is tested in an industrial application by assessing the steelmaking time, dephosphorization rate, and mass fraction of FeO in the resulting slag.

## 2. Structural Design and Validation

**Figure 1:**
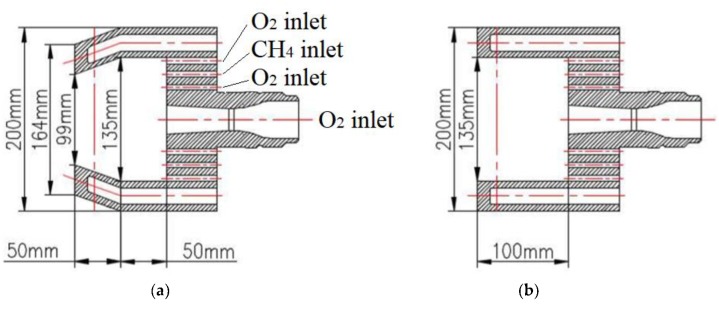

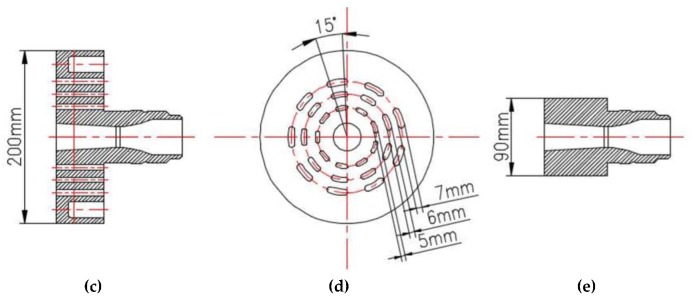
Cross-sectional and front view of the coherent lances with various structures. (**a**) The cross-sectional for a flap restricted coherent lance; (**b**) cross-sectional for an open restricted coherent lance; (**c**) cross-sectional for a conventional coherent lance; (**d**) front view of the whole coherent lances; (**e**) cross-sectional for a conventional lance.

The Figure 1 showed three kinds of coherent lances and one kind of conventional supersonic oxygen lance. For the three kinds of coherent lance structures, the positioning of their arrangements for main oxygen and shrouding nozzles were same, as shown in Figure 1d. The key distinction between them was the shape of the restriction structure for the coherent lance tip. Hereafter, the coherent lance depicted in Figure 1a,b would be referred to as a flap and an open restriction structure coherent lance, respectively, or simply as a ‘flap coherent lance’ and ‘open coherent lance’ for short, respectively. The coherent lance depicted in Figure 1c,e would be addressed as a conventional coherent lance and a conventional lance, respectively.

The design Mach number of the main oxygen flow was 2.0, with a throat diameter of 24.6 mm and an exit diameter of 31.8 mm. The shrouding nozzles were respectively distributed around three concentric circles, as presented in Figure 1. The inner and outer nozzles were injected an O_2_ flow, and the intermediate nozzle injected a CH_4_ flow. The diameter of these concentric rings, from inside to outside, were 34.0, 49.5, and 128.0 mm, respectively. The exactly same nozzle parameters were applied in both combustion experiment and the numerical simulation. The previous literatures have provided the details of the experimental equipment and its control process used in this paper [12,13,14], and only a briefly explanation has been provided in this paper.

The pitot tube and thermocouple were fixed at specific locations to measure the pressure and the static temperature of the main oxygen jet. The pitot tube was designed with a water-cooled structure. When the dynamic and static pressure were measured by the pitot tube, the Equations (1) and (2) were used to calculate the axial velocity of the coherent jet [18]

(1) $$V_{axial}=\sqrt{\frac{2RT\gamma }{\gamma -1}\left[{\left(\frac{p_{0}}{p}\right)}^{(\gamma -1)/\gamma }-1\right]},\;\mathrm{Ma}>0.3$$

(2) $$V_{axial}=\sqrt{\frac{2(p-p_{0})}{\rho }},\;\mathrm{Ma}<0.3$$

where, *p* and *p_0_* are the dynamic and static pressure of the supersonic jet, respectively. *V_axial_* and *ρ* are the axial velocity and density of the gas flow. *R* and *T* are ideal gas constant and static temperature, respectively. *γ* is the ratio of oxygen specific heat being 1.4.

## 3. Numerical Simulation

### 3.1. Governing Equations

In this paper, the following governing equations were used for the simulation process to integrate the Reynolds-averaged Navier–Stokes (RANS) [19]:

Continuity equation:

(3) $$\frac{\partial \rho }{\partial t}+\nabla (\rho \vec{v})=0$$

Momentum equation:

(4) $$\frac{\partial }{\partial x_{j}}(\rho v_{i})+\nabla (\rho \vec{v}\vec{v})=-\nabla P+\nabla [\mu (\nabla \vec{v})]+\rho \vec{g}$$

Energy equation:

(5) $$\frac{\partial (\rho E)}{\partial t}+\nabla [\vec{v}(\rho E+\rho )]=\nabla (k_{eff}\nabla T-\sum_{j}h_{i}\vec{J_{i}}+(\vec{\tau_{eff}}\cdot \vec{v}))+S_{h}$$

where, *ρ* and *µ* are the density and kinetic viscosity for fluid, respectively; $\vec{J_{i}}$ and *h_i_* are the diffusion flux and enthalpy of species i; $\vec{v}$ is the instantaneous velocity. *k_eff_* is the effective thermal conductivity; *S_h_* represents the heat of chemical reaction; *R* and $\vec{g}$ are the ideal gas and acceleration of gravity constant being 8.31 and 9.81, respectively; *E* is the total energy.

When the main oxygen jet passed through the Laval nozzle, its high pressure energy was transformed into its kinetic energy, and then its temperature and density also changed. Thus, the gas phases should be defined as the ideal gas for the simulation process, and their parameters could be calculated by the equation [20]

(6) $$\rho =\frac{PM}{nRT}$$

where, *M*, *P*, and *n* are the mass, total pressure, and mole number of the gas, respectively.

The realizable *k*-*ε* turbulence model was adopted to calculate the simulation data. The following equations were used to calculate turbulence kinetic energy (*k*) which was used to construct its dissipation rate (*ε*) [19]

(7) $$\frac{\partial }{\partial x_{i}}(\rho ku_{i})=\frac{\partial }{\partial x_{j}}[(\mu +\frac{\mu_{t}}{\sigma_{\kappa }})\frac{\partial k}{\partial x_{i}}]-G_{k}+G_{b}-\rho \varepsilon -Y_{M}$$

(8) $$\frac{\partial (\rho \varepsilon u_{i})}{\partial x_{i}}=\frac{\partial }{\partial x_{j}}\left(\mu +\frac{\mu_{t}}{\sigma_{\varepsilon }}\frac{\partial \varepsilon }{\partial x_{i}}\right)+\rho C_{1\varepsilon }S_{\varepsilon }-\rho C_{2\varepsilon }\frac{\varepsilon^{2}}{\kappa +\sqrt{u\varepsilon }}+C_{1\varepsilon }\frac{\varepsilon }{\kappa }C_{3\varepsilon }G_{b}$$

where, *C_1__ε_*, *C_2__ε_*, *C_3__ε_*, *σ_k_*, and *σ_ε_* are the constants, with values of 1.44, 1.92, 0.09, 1.0, and 1.2, respectively [19,21]. *G_k_* and *G_b_* are the generation of turbulence kinetic energy due to the mean velocity gradient and buoyancy. *Y_M_* is the contribution of the fluctuating dilatation in the compressible turbulence process, as defined by the equation

(9) $$Y_{M}=2\rho \varepsilon \frac{k}{\gamma RT}$$

The eddy dissipation concept (EDC) model with the multi-step chemical kinetic mechanisms (GRI Mech, 3.0, c/o Gas Research Institute, Chicago, IL, USA) was used to simulate the combustion reaction. The transport equation of mass fraction for each species in the chemical mechanism could be presented as [22]:

(10) $$\frac{\partial }{\partial t}(\rho Y_{i})+\nabla (\rho \vec{v}Y_{i})=-\nabla \vec{J_{i}}+R_{i}$$

where *Y_i_* is the local mass fraction of each species i, and *R_i_* is the net rate of species i production by chemical reaction, and *J_i_* is the diffusion flux of each species i.

### 3.2. Simulation Details

**Figure 2:**
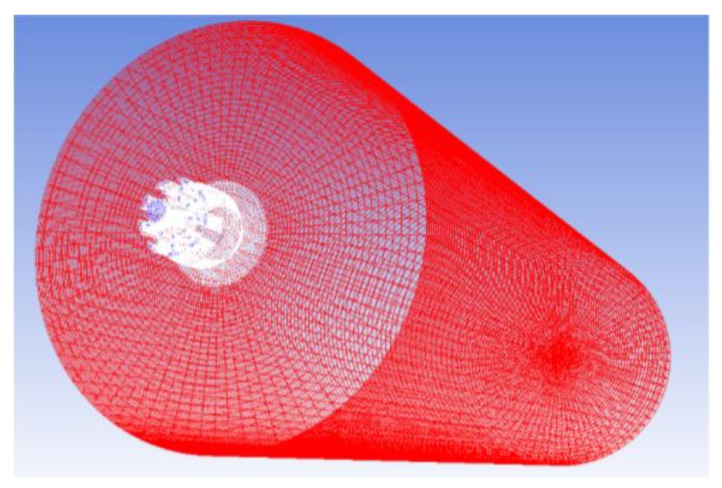
Structure grid of the computational domain.

Figure 2 depicted a 3D computational domain grid of the flap coherent lance used in this study. In this research, all the cell structures were used hexahedral mesh, and its orthogonal quality (checked in the Fluent) was 8.9–9.1. The main oxygen jet and shrouding jet flowing zones were used as the computational domain, and the exit diameter of the main oxygen Laval nozzle was defined as 1 De. The main computational domain ranged from the tip of the Laval nozzle to 85 De downstream in an axial direction and 12.5 De in a radial direction. Under the initial condition, the air filled the whole computational domain. Table 1 and Table 2 showed the boundary conditions and the gas thermophysical properties used in the simulation model, respectively.

A pressure-based solver using a steady-state model was employed to calculate the RANS equation. The standard spatial discretization approach was used to calculate pressure values, and other variables, including energy, turbulent kinetic energy, and dissipation rate were solved using a QUICK (Quadratic Upstream Interpolation for Convective Kinematics) scheme. The walls were defined as the standard wall at the non-slip condition. The discrete ordinate (DO) model was considered for radiation phenomenon to achieve noticeable effects on the results. Moreover, the weighted sum of gray gas (WSGG) model was used to calculate the total emissivity of mixture gases as the function of their temperatures and pressures.

Solution convergence for the numerical model were determined when the energy residual was <10^−7^ and residuals for other variables was <10^−^^5^, with consecutive iterations for temperature and velocity at the outlet of the computational domain being <1.0 K and 1.0 m/s, respectively.

### 3.3. Mesh Independency Test

**Figure 3:**
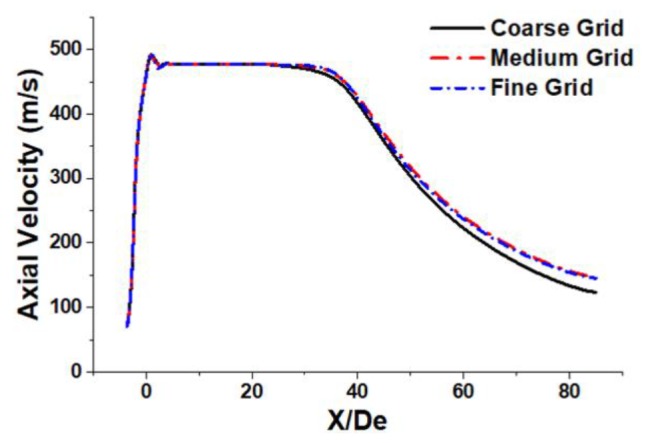
Axial velocity distribution of the coherent jets using different mesh levels.

To insure the accuracy of simulation data, the grid independency of the simulation model has been tested using three kinds of grid levels: coarse grid (322,643 cells), medium grid (616,837 cells), and fine grid (856,715 cells). Figure 3 depicted the axial velocity distributions of the coherent jets at the jet centerline, using those grid levels.

Figure 3 showed that the axial velocity variation between the coarse and the medium grid was found to be 19.1% X/De. This variation between the medium and the fine grid was found to be <1%, which means that the simulation was not sensitive to their physical properties. The computational time required to calculate using the fine grid was approximately 2.1 times greater than the time required to calculate using the medium grid level, and as a consequence the medium grid was chosen for further studies.

## 4. Results and Discussion

### 4.1. Velocity Profile

**Figure 4:**
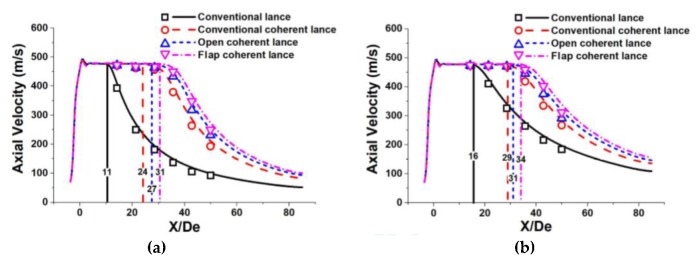
Axial velocity distribution of the coherent jets using various restriction structures at room and high ambient temperature: (**a**) at an ambient temperature of 300 K; (**b**) at an ambient temperature is 1700 K.

Figure 4 shows the axial velocity profiles of coherent jets using various restriction structures at different ambient temperatures. The simulation results are shown by solid and dotted lines, whilst the measurement data is represented by various symbols (□, ○, △, and ▽). Room and high ambient temperature are defined as 300 K and 1700 K, respectively. The results show that the experimental data is in a good agreement with the simulation results.

The velocity potential core length of the main oxygen jet at room ambient temperature using a conventional coherent lance, an open coherent lance and a flap coherent lance were 24 De, 27 De, and 31 De, respectively. At a high ambient temperature, the velocity potential core length of the main oxygen jet using a conventional coherent lance, an open coherent lance and a flap coherent lance were 29 De, 31 De, and 34 De, respectively. For the comparison, the velocity potential core length of the main oxygen jet using a conventional lance at room and high ambient temperature were 11 De and 16 De, respectively. The average potential core lengths formed by a coherent lance with a restriction structure were 1.2- and 2.3-times larger than those generated by a conventional coherent lance and a conventional lance, respectively. Thus, it can be concluded that a coherent lance with a restriction structure is able to prolong the velocity potential core length of the main oxygen jet. The coherent lance with a flap restriction structure is able to further improve this trend, when compared to an open restriction structure.

The length of the velocity potential core for the main oxygen jet at the high ambient temperature using a conventional lance, a conventional coherent lance, an open coherent lance and a flap coherent lance were 1.45-, 1.21-, 1.15-, and 1.10-times longer than those were at room ambient temperature respectively. Hence, although a higher ambient temperature will generally prolong the potential core length, it has the greatest impact upon a conventional lance and the smallest impact upon a flap coherent lance.

To assess the effect the axial velocity of the shrouding flame, an annulus was created in the simulation model. The annulus was located 100 mm in an axial direction from the exit of the main oxygen Laval nozzle and had a maximum and minimum radius of 46.0 mm and 33.8 mm, respectively. The average static temperature and axial velocity of the gas flow over this annulus at room and high ambient temperature using different coherent lances are shown in Table 3. The results show the average static temperature of the gas flow over the annulus at room and high ambient temperature was much higher than the inlet temperature of the main oxygen jet (298 K). This proves that the gas flow over the annulus is the shrouding flame, which is not the main oxygen jet. The results also show that a restriction structure is able to increase the axial velocity of the shrouding flame, comparedwith the conventional coherent lance. At the same time, a flap restriction structure also further increases this tendency.

### 4.2. Static Temperature Profile

**Figure 5:**
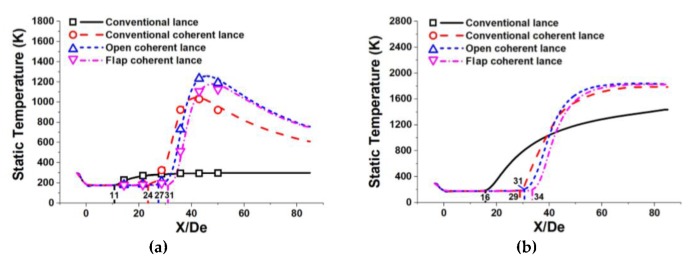
Static temperature distribution of the coherent jets using various restriction structure at room and high ambient temperature: (**a**) at an ambient temperature of 300 K; (**b**) at an ambient temperature of 1700 K.

Figure 5 represents the static temperature profiles of the coherent jets using the various restriction structures at different ambient temperatures. In this study, the static temperature of the oxygen jet was only measured at the room ambient temperature, because the thermocouple might be damaged during the furnace heating process.

During the main oxygen jet passes through the Laval nozzle, its pressure and thermal energy would be transferred into kinetic energy, resulting in its static temperature reducing. Then the main oxygen jet would be heated by the ambient temperature or the high-temperature flame at the end of the potential core, which makes its static temperature improve. At last, the static temperature of the main oxygen jet asymptotically approaching the ambient temperature, as shown in Figure 5.

The point at where the static temperature of a coherent jet starts increasing can be defined as the temperature increasing point, TIP for short. The location for the TIP using a conventional coherent lance, an open coherent lance and a flap coherent lance were 24 De, 27 De, and 31 De, respectively. At a high ambient temperature, the location for the TIP using a conventional coherent lance, an open coherent lance and a flap coherent lance were 29 De, 31 De, and 34 De, respectively. Hence, the location for the TIP was very close to the location for the end point of velocity potential core for the main oxygen jet. With no entrainment effect between the main oxygen jet and the ambient gas, the total energy of the main oxygen jet at the centerline did not change, making its static temperature stable. When the main oxygen jet reaches the end of potential core, it will absorb the heat energy from the flame, because of their relative temperature gradients, making its static temperature improve. As the main oxygen jet fully develops, its static temperature will gradually become equal to the ambient temperature.

As shown in Figure 5, the maximum static temperature of the main oxygen jet at room ambient temperature using a conventional coherent lance, an open coherent lance and a flap coherent lance were 1047 K, 1258 K, and 1180 K in an axial direction of 42 De, 46 De, and 48 De, respectively. For the high ambient condition, the maximum static temperature of the main oxygen jet at room ambient temperature using a conventional coherent lance, an open coherent lance and a flap coherent lance were 1410 K, 1610 K, and 1512 K in an axial direction of 45 De, 48 De, and 50 De, respectively. Hence, a coherent lance using a restriction structure is able to extend the high-temperature zone of the flame in the axial direction, when compared to a conventional coherent lance. However, the open coherent lance achieves a higher maximum static temperature of main oxygen jet and also generates a shorter potential core, when compared to the flap coherent lance. When the shrouding jet passes through the flap restriction structure, some of its heat energy of the shrouding flame is transformed into its kinetic energy, resulting in suppressing its maximum static temperature. This energy transformation resembles in some respects the behavior of the main oxygen jet, as the main oxygen jet passes through the Laval nozzle. As a result, the maximum static temperature of a coherent jet using a flap restriction structure is lower than that using an open restriction structure.

**Figure 6:**
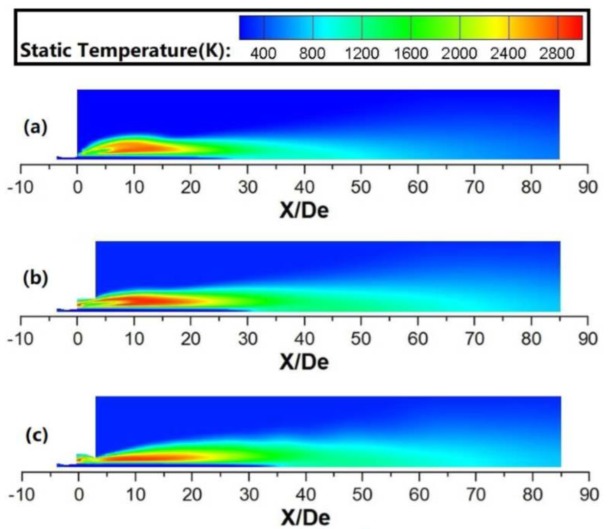
Static temperature profiles for various coherent jets at room ambient temperature: (**a**) conventional coherent lance; (**b**) open coherent lance; (**c**) flap coherent lance.

**Figure 7:**
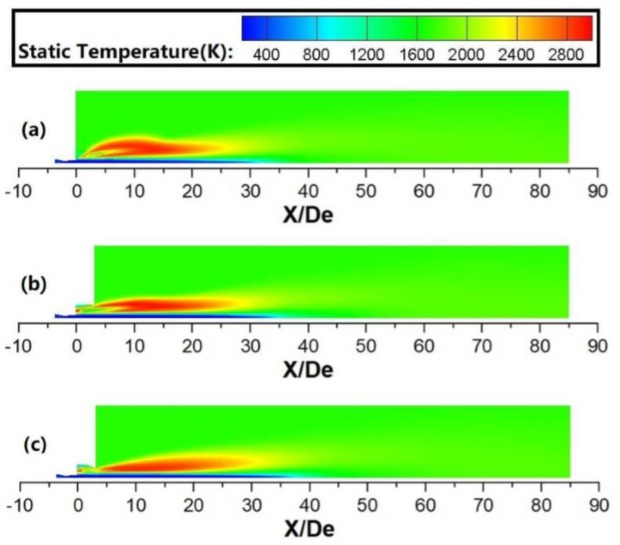
Static temperature profiles for various coherent jets at high ambient temperature: (**a**) conventional coherent lance; (**b**) open coherent lance; (**c**) flap coherent lance.

As shown in Figure 6 and Figure 7, the shrouding flame would rapidly expand after it passed through the shrouding nozzles, and a restriction structure can suppress this tendency. The results show that the energy of the shrouding flame is transported in both axial and radial directions. In this research, the total energy value using various coherent lances is the same, because the flow rate of the shrouding O_2_ and CH_4_ is unchanged for any cases. A restriction structure can delay the energy transmission in the radial direction at the coherent lance tip, resulting in more heat energy transmission in the axial direction, which prolongs the high-temperature zone of shrouding jet in the axial direction. During this process, the main oxygen jet using a restriction structure generates more heat energy from the shrouding flame, which makes its static temperature higher, as shown in the Figure 5.

### 4.3. Dynamic Pressure Profile

**Figure 8:**
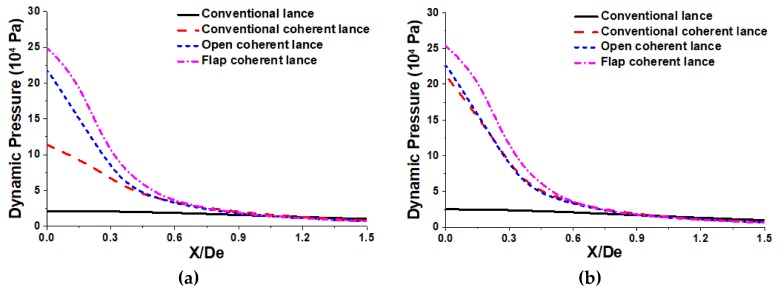
Dynamic pressure profiles of the coherent jets using various restriction structures at room and high ambient temperature: (**a**) at an ambient temperature of 300 K; (**b**) at an ambient temperature of 1700 K.

As a major factor in the shape of the impaction cavity of the molten bath, the profiles of the dynamic pressure of coherent jets using various restriction structures at different ambient temperatures are showed in the Figure 8. In this paper, the dynamic pressure profile of the coherent jet at a centerline of X/De = 32 has been selected, based on the distance between the lance tip and molten bath.

At the ambient room temperature, the maximum dynamic pressure value of the coherent jet using a conventional lance, a conventional coherent lance, an open coherent lance and a flap coherent lance, were 2.1 × 10^4^, 11.4 × 10^4^, 21.9 × 10^4^, and 25.0 × 10^4^ Pa, respectively. At the high room temperature, the maximum dynamic pressure value of the coherent jet using a conventional lance, a conventional coherent lance, an open coherent lance and a flap coherent lance, were 2.5 × 10^4^, 21.3 × 10^4^, 22.7 × 10^4^, and 25.4 × 10^4^ Pa, respectively. The results show the maximum dynamic pressures of the coherent jet at the high ambient temperature, using a conventional lance, a conventional coherent lance, an open coherent lance and a flap coherent lance, were 1.19, 1.87, 1.04, and 1.02 times larger than for the coherent jet at the room ambient temperature, respectively. Therefore, increasing the ambient temperature could improve the maximum dynamic pressure. Besides, the ambient temperature has the largest and smallest effect on the maximum dynamic pressures of the coherent jet using a conventional coherent lance and a flap coherent lance, respectively.

As mentioned in the preview literatures [9,23,24], a supersonic jet with higher dynamic pressure can form bigger depth and radius of impaction cavity. Based on the result, the dynamic pressure variation using the different lance structures is noticeable in the range from 0 to 0.9 De in the radius direction, as presented in Figure 8, and those areas of field under the curve-line are calculated. When the ambient temperature is 300 K, the integration areas using a conventional lance, a conventional coherent lance, an open coherent lance and a flap coherent lance, were 1.7 × 10^4^, 5.0 × 10^4^, 6.7 × 10^4^, and 8.2 × 10^4^ Pa, respectively. When ambient temperature is 1700 K, the integration areas using a conventional lance, a conventional coherent lance, an open coherent lance and a flap coherent lance, were 2.0 × 10^4^, 6.6 × 10^4^, 7.0 × 10^4^, and 8.5 × 10^4^ Pa, respectively. The flap coherent lance can form the biggest impaction cavity, next the open coherent lance, the conventional coherent lance is in the third place, and the conventional lance is the smallest both at room and high ambient temperature. Therefore, the flap coherent lance can further improve the surface area between the supersonic jet and molten bath, which accelerates the dephosphorization and decarburization rate in the steelmaking process.

### 4.4. Industrial Application

According to the results of the combustion experiment and the numerical simulation research, the coherent lance using a flap restriction structure achieves greater impaction ability than the other coherent lance structures. In order to confirm the metallurgical and operational effects for this coherent lance structure, both the flap and conventional coherent lances were tested in a 75 t electrical arc furnace. The data from 160 heats was collected during the smelting process for each kind of coherent lance, with the control processes remaining identical for each heat. For the purposes of comparative analysis, we collected data including the steelmaking time, the dephosphorization rate and the mass fraction of FeO in the slag.

The conditions relating to liquid iron (prior to the steelmaking process) and molten steel (after the steelmaking process) are shown in Table 4. For the smelting with each of the different coherent lances, the initial conditions for the liquid iron were fundamentally the same, which means that the initial conditions had no impact on the results of the industrial application. When compared to a conventional coherent lance, the average [C][O] and steelmaking time when using a flap coherent lance were reduced by 7.7% and 1.2%, respectively. This proves that the impaction ability of a flap coherent jet is better than the conventional coherent lance structure. It also confirms the accuracy of the previous combustion experiment and numerical simulation results.

**Figure 9:**
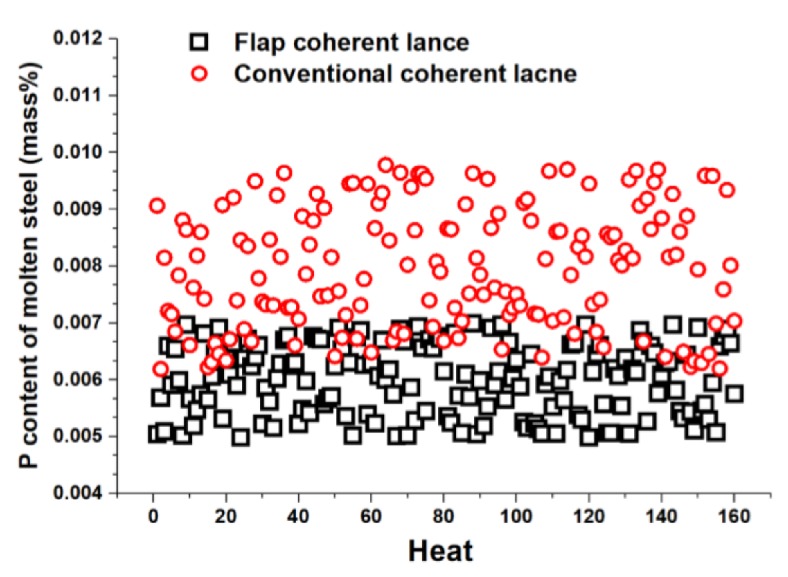
P content distribution of molten steel for the two kinds of coherent lances.

Figure 9 presents the distributions of the phosphorus in the molten steel using two kinds of coherent lances. The results show that the content of phosphorus content in the molten steel had a distribution of 0.005 to 0.007 wt % using the flap coherent lance and 0.006 to 0.010 wt % using the conventional coherent lance. The average phosphorus contents for flap and conventional coherent lance were 0.006 and 0.008 wt %, respectively. Thus, both the average value and the range of distribution for P content using a flap coherent lance were lower than those using a conventional coherent lance. This proves that a flap coherent lance offers better control performance.

**Figure 10:**
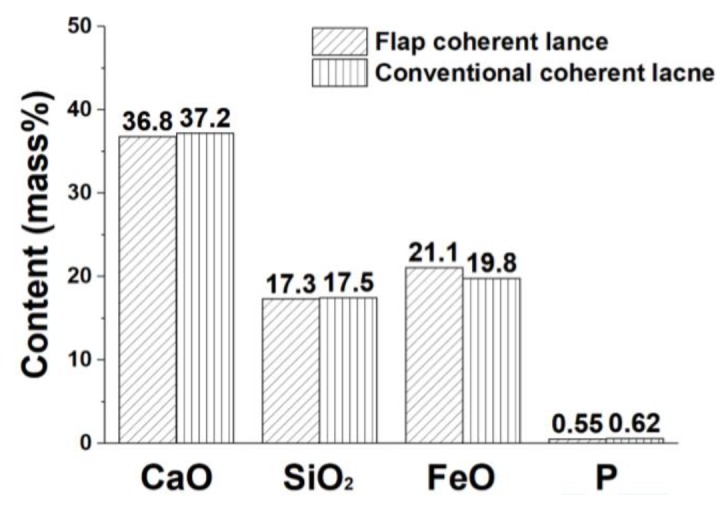
Distribution of the average CaO, SiO_2_, FeO, and P content in the liquid slag.

Figure 10 shows the profiles of the average CaO, SiO_2_, FeO, and P content in the end-point slag after the testing with the different lances. As the same slag basicity being 2.1, the average FeO content using a flap coherent lance was 1.3 wt % lower in the end-point slag than that using a conventional coherent lance. That means a flap coherent lance is beneficial to improvements in the metal yield rate. Moreover, the average P content was increased by 0.07 wt % using the flap coherent lance, due to a bigger dephosphorization rate.

**Figure 11:**
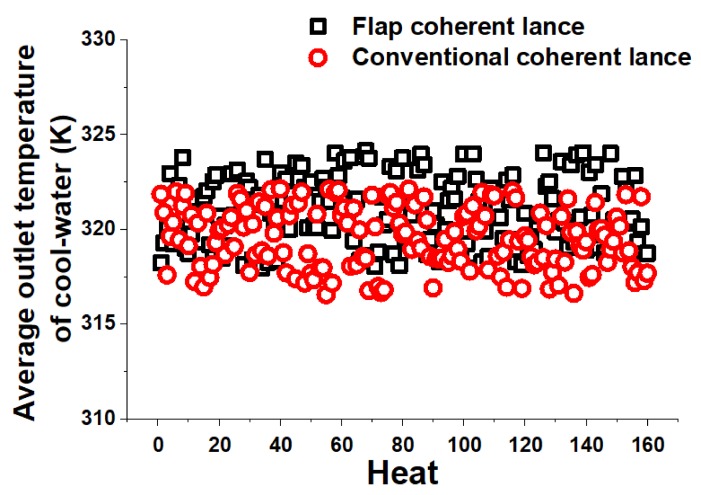
Average outlet temperature of cool-water profiles for the two kinds of coherent lances.

Figure 11 presents the average outlet temperature of cool-water profiles for two kinds of coherent lances in steelmaking process. When the flow rate of cool-water for two kinds of coherent lances were both 15.0 t/h, the average temperature of cool-water had a distribution of 318.0 K (44.8 °C) to 324.2 K (51.0 °C) using the flap coherent lance and 316.6 K (43.4 °C) to 322.2 K (49.0 °C) using the conventional coherent lance, as shown in the Figure 11. The average temperature of cool-water for flap and conventional coherent lance were 321.0 K (47.8 °C) and 319.6 K (46.4 °C), respectively.

As depicted in the Table 4, the average condition for liquid iron is same for both the flap and conventional coherent lance. That means the initial total energy generated by the oxidation reactions between the oxygen gas and elements in the molten bath (carbon, phosphorus, and silicon). The heat productivity per unit time formed by the flap coherent lance is bigger than that by the conventional coherent lance, by comparing the steelmaking time. The thermal radiation of molten bath is stronger using the flap coherent lance, which makes its average outlet temperature of cool-water higher.

After the 50th heat, the surface structures of two kinds of coherent lances have no significant difference, comparing with their initial status. Then, the flow rate of cool-water for the flap coherent lance has been increased to 15.5 t/h, for eliminating the average outlet temperature of cool-water using two kinds of coherent lances. In this case, the flap coherent lance is still working properly after 1000 heats, and meets the requirement of its service life, according to the production safety standards proposed by the plant.

Based on the industrial application result, the impaction ability of coherent jet using a flap coherent lance is better than that using a conventional coherent lance, which is consistent with the results of the combustion experiment and numerical simulation.

## 5. Conclusions

In this research, the flow fields for coherent jets using various kinds of restriction structure were investigated at room and high ambient temperatures. Both numerical simulation and combustion experiments were undertaken, and the data generated by each of them was in good agreement. The main conclusions can be summarized as follows:

(1) The average potential core length for the main oxygen jet formed by a coherent lance using a restriction structure was 1.2 and 2.3 times larger than that formed by a conventional coherent lance or a conventional lance, respectively. Therefore, a coherent lance with a restriction structure is able to prolong the velocity potential core length for the main oxygen jet, and a flap restriction structure further improves upon this trend.
(2) When compared to a conventional coherent lance, a coherent lance using a restriction structure is able to delay energy transmission in a radial direction at the coherent lance tip, which enlarges the high-temperature zone in a axial direction and increases the axial velocity of the shrouding flame. For coherent jets using a flap restriction structure, part of its heat energy of the shrouding flame is transformed into its kinetic energy, when the shrouding flame passes through the coherent lance tip. As a result, the maximum temperature of the main oxygen jet using a flap restriction structure is lower than that using an open restriction structure.
(3) Comparison to all the coherent lance structures, the flap coherent lance can increase the dynamic pressure of the supersonic jet, which further improves the surface area between the supersonic jet and molten bath, resulting in accelerating the dephosphorization and decarburization rates in the steelmaking process. Besides, the ambient temperature has the largest and smallest effect on the maximum dynamic pressures of the coherent jet using conventional coherent lance and flap coherent lances, respectively.
(4) During the 75 t EAF steelmaking process, the average [C][O] and steelmaking time using a flap coherent lance was reduced by 7.7% and 1.2%, respectively, and the average FeO content dropped by 1.3 wt % in the end-point slag, whilst the average dephosphorization rate increased by 1.6%, when compared to a conventional coherent lance. This is consistent with the results of the combustion experiment and the numerical simulation.

## Figures and Tables

**Table 1:** Boundary conditions.

Name of Boundary	Type of Boundary Conditions	Values
Main oxygen inlet	Mass flow rate	0.9921 kg/s
	Mass fractions	O_2_ = 100%
	Oxygen temperature	298 K
Shrouding gas inlet (O_2_)	Mass flow rate	0.1190 kg/s
	Mass fractions	O_2_ = 100%
	Gas temperature	298 K
Shrouding gas inlet (CH_4_)	Mass flow rate	0.0595 kg/s
	Mass fractions	CH_4_ = 100%
	Gas temperature	298 K
Outlet	Static pressure	101325 Pa
	Mass fractions	O_2_ = 23%, N_2_ = 77%
	Ambient temperature	300 K, 1700 K

**Table 2:** Thermo-physical properties for gases.

	O_2_	CH_4_	Air
Density/(kg·m^−3^)	Ideal gas	Ideal gas	Ideal gas
Cp/(J·kg^−1^·K^−1^)	Piecewise-polynomial	Piecewise-polynomial	Piecewise-polynomial
Molecular weight/(kg·kgmol^−1^)	31.999	16.043	28.966
Standard state enthalpy/(J·kgmol^−1^)	0	−7.490 × 10^7^	-
Standard state entropy/(J·kgmol^−1^·K^−1^)	2.050 × 10^5^	1.864 × 10^5^	1.934 × 10^5^

**Table 3:** Average static temperature and axial velocity for various coherent lances.

Label		Conventional Coherent Lance	Open Coherent Lance	Flap Coherent Lance
Room ambient temperature	Average static temperature (K)	915	1482	1934
	Average axial velocity (m/s)	64	107	223
High ambient temperature	Average static temperature (K)	1298	1434	1646
	Average axial velocity (m/s)	94	167	300

**Table 4:** Average conditions for liquid iron and molten steel.

Label	Liquid Iron			Molten Steel				Steelmaking Time (min)
	C (%)	P (%)	Temperature (K)	C(%)	P(%)	[C][O] (10^−4^)	Temperature (K)	
Flap coherent lance	3.57	0.132	1558	0.076	0.006	0.0048	1883	49.5
Conventional coherent lance	3.57	0.131	1559	0.077	0.008	0.0052	1882	50.1
